# RANTES/CCL5 mediated-biological effects depend on the syndecan-4/PKCα signaling pathway

**DOI:** 10.1242/bio.20148227

**Published:** 2014-09-26

**Authors:** Loïc Maillard, Naoaki Saito, Hanna Hlawaty, Véronique Friand, Nadine Suffee, Fanny Chmilewsky, Oualid Haddad, Christelle Laguillier, Erwan Guyot, Takehiko Ueyama, Olivier Oudar, Angela Sutton, Nathalie Charnaux

**Affiliations:** 1 Inserm U1148, Laboratory for Vascular Translational Science, Bio-ingénierie Cardio-vasculaire, UFR SMBH, Université Paris 13, Sorbonne Paris Cité, 74 rue Marcel Cachin, 93017 Bobigny, France; 2 Laboratory of Molecular Pharmacology, Biosignal Research Center, Kobe University, Kobe 657-8501, Japan; 3 Laboratoire de Biochimie, Hôpital Jean Verdier, AP-HP, 93143 Bondy, France

**Keywords:** Syndecan-4, Chemokine, PKC, RANTES/CCL5, Endothelial cell

## Abstract

The perpetuation of angiogenesis is involved in certain chronic inflammatory diseases. The accelerated neovascularisation may result from an inflammatory status with a response of both endothelial cells and monocytes to inflammatory mediators such as chemokines. We have previously described *in vitro* and *in vivo* the pro-angiogenic effects of the chemokine Regulated on Activation, Normal T Cell Expressed and Secreted (RANTES)/CCL5. The effects of RANTES/CCL5 may be related to its binding to G protein-coupled receptors and to proteoglycans such as syndecan-1 and -4. The aim of this study was to evaluate the functionality of syndecan-4 as a co-receptor of RANTES/CCL5 by the use of mutated syndecan-4 constructs. Our data demonstrate that site-directed mutations in syndecan-4 modify RANTES/CCL5 biological activities in endothelial cells. The SDC4S179A mutant, associated with an induced protein kinase C (PKC)α activation, leads to higher RANTES/CCL5 pro-angiogenic effects, whereas the SDC4L188QQ and the SDC4A198del mutants, leading to lower phosphatidylinositol 4,5-bisphosphate (PIP_2_) binding or to lower PDZ protein binding respectively, are associated with reduced RANTES/CCL5 cellular effects. Moreover, our data highlight that the intracellular domain of SDC-4 is involved in RANTES/CCL5-induced activation of the PKCα signaling pathway and biological effect. As RANTES/CCL5 is involved in various physiopathological processes, the development of a new therapeutic strategy may be reliant on the mechanism by which RANTES/CCL5 exerts its biological activities, for example by targeting the binding of the chemokine to its proteoglycan receptor.

## INTRODUCTION

A member of the β-chemokine family, the CC-chemokine Regulated upon Activation, Normal T-cell Expressed and Secreted (RANTES)/CCL5 is both a T cell chemoattractant and an immunoregulatory molecule. It is now apparent that RANTES/CCL5 exhibits critical functions in many diverse physiopathological mechanisms, including tumor progression and angiogenesis (Suffee et al., 2011; Rossi and Zlotnik, 2000; Soria and Ben-Baruch, 2008). Indeed, we have previously demonstrated that RANTES/CCL5 is pro-angiogenic in rat in a subcutaneous model (Suffee et al., 2012). This activity is related to the *in vitro* promotion of endothelial cell migration, spreading and neo-vessel formation. RANTES/CCL5 signals through its specific G Protein-Coupled Receptors (GPCRs) CCR1, CCR3 and CCR5. Moreover, RANTES/CCL5, like other chemokines, also binds to glycosaminoglycans (GAGs), which are long, linear, and heterogenous sulfated polysaccharides. RANTES/CCL5 exhibits selectivity in glycosaminoglycan binding with the highest affinity (nanomolar range) for heparin (Martin et al., 2001; Proudfoot et al., 2001). Glycosaminoglycans exist in covalent linkage to a protein core as proteoglycans. We have previously demonstrated that RANTES/CCL5 not only associates with its GPCRs but also with heparan sulfate proteoglycan belonging to the syndecan family, syndecan-1 (SDC-1) and syndecan-4 (SDC-4) on various cell types (Sutton et al., 2007; Charni et al., 2009; Slimani et al., 2003a; Slimani et al., 2003b). The binding of the chemokine to glycosaminoglycan chains modulate RANTES/CCL5 biological activities. Indeed, soluble heparin, GAG mimetics or GAG-binding deficient mutants of RANTES/CCL5 can modulate the biological activities of the chemokine as shown *in vitro* (Charni et al., 2009; Sutton et al., 2007) or *in vivo* (Suffee et al., 2012; Nellen et al., 2012).

Syndecan-4 (SDC-4) is one of a family of four transmembrane heparan sulfate proteoglycans, whose extracellular domains interact with various soluble and insoluble factors in the extracellular matrix (ECM). Syndecans have been thought to act as co-receptors for various heparin-binding growth factors such as fibroblast growth factors (FGFs), vascular endothelial growth factors (VEGFs) and fibronectin-binding integrins (Kwon et al., 2012; Beauvais and Rapraeger, 2010; Bernfield et al., 1999). An evolutionary conserved cytoplasmic domain on syndecans supports a key role for cell surface ligand binding and cytoplasmic signaling. Common to all syndecans, three regions of cytoplasmic domain have been identified. The first (C1) is the membrane-proximal region that binds Src kinase, ezrin, and cortactin (Granés et al., 2003; Kinnunen et al., 1998). The second (C2) is a C-terminal region that contains a post-synaptic density 95, discs-large, ZO-1 (PDZ)-domain binding motif (Multhaupt et al., 2009). The variable (V) domain is located between the two conserved domains and its sequence is unique to each syndecan family member. The V domain of SDC-4 binds to phosphatidylinositol 4,5-bisphosphate (PIP_2_) and also to protein kinase Cα (PKCα) complex, α-actinin, and syndesmos (Lim et al., 2003; Horowitz et al., 1999; Greene et al., 2003; Denhez et al., 2002). These interactions are responsible for the previously demonstrated SDC-4 role in cytoskeleton regulation that includes formation of focal adhesions, of dynamic stress fibers, and cell protrusions (Kwon et al., 2012). SDC-4 null mice are viable and fertile but exhibit defective skin wound healing reflecting impaired cell migration and angiogenesis (Echtermeyer et al., 2001; Okina et al., 2012).

Therefore, the hypothesis tested here is that the interaction of RANTES/CCL5 with SDC-4 triggers the transduction of signals leading to changes in the intracellular environment. To that purpose, we will evaluate the involvement of intracellular cytoplasmic SDC-4 domains in RANTES/CCL5-induced angiogenesis.

## RESULTS

### Site-directed mutations in syndecan-4 modify RANTES/CCL5 biological activities in endothelial cells

**Fig. 1.**
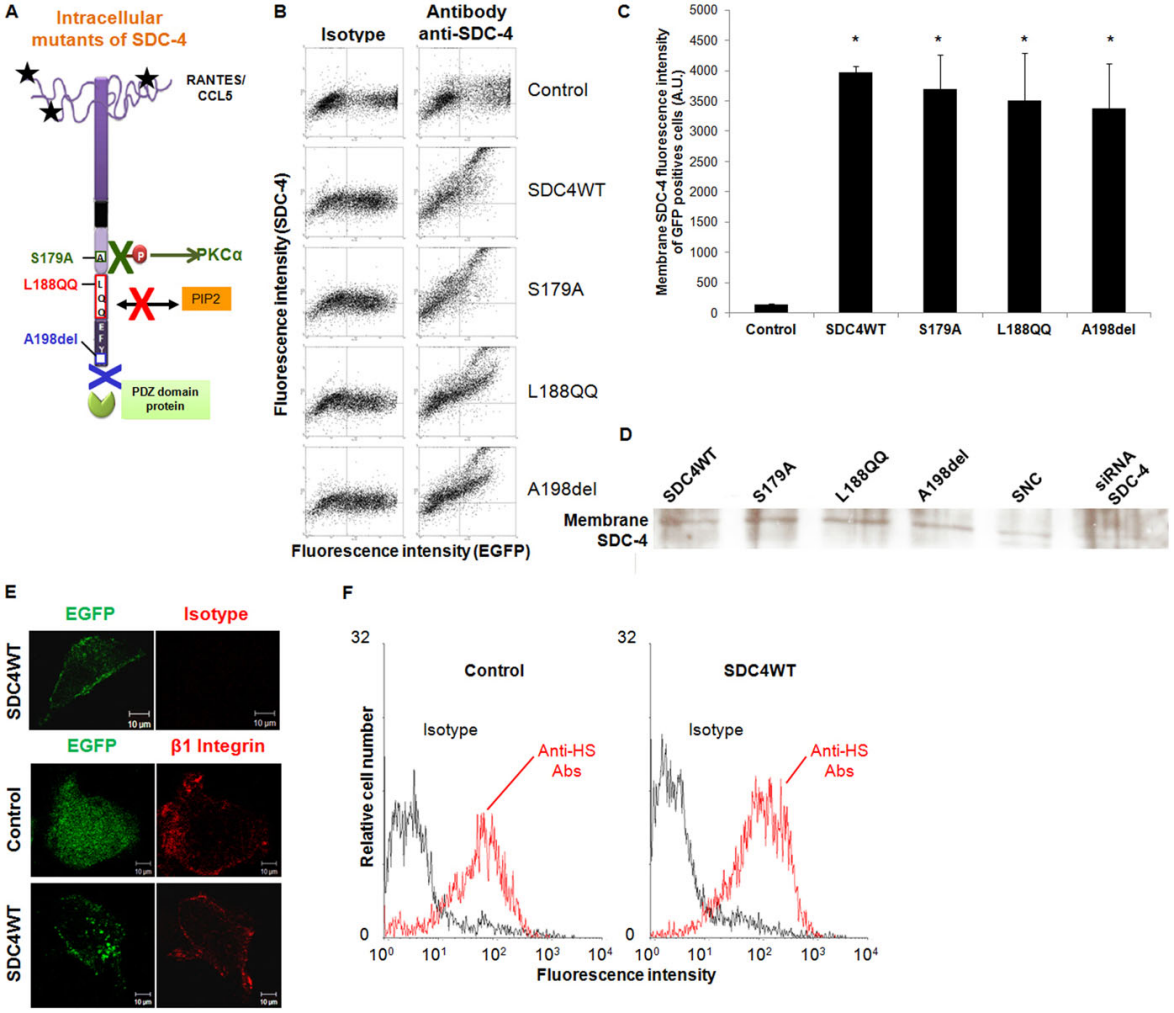
Syndecan-4 mutants are located at the membrane of endothelial cells. (A) Schemes of the syndecan-4 cytoplasmic constructs used in the study: S179A mutation expected to lead to a constitutive PKCα activation; PIP_2_^−^ (Y188KK to L188QQ) mutation; PDZ^−^ (deletion of the COOH-terminal A198 residue) mutation. (B) HUV-EC-C transfection efficiency was determined by flow cytometry on non-permeabilized cells. Transfected cells were quantified for EGFP fluorescence (horizontal axis) and for fluorescent SDC-4 staining after cell incubation with specific antibody or control isotype (vertical axis). Transfection rate from representative experiments were estimated for each plasmid. (C) Cells were transfected with GFP plasmid (control), SDC4WT-GFP (SDC4WT) or SDC-4 constructs (S179A, L188QQ or A198del) (horizontal axis). Expression of membrane SDC-4 in GFP-positive cells was quantified by flow cytometry with specific anti-SDC-4 antibodies without cell permeabilization (vertical axis). * P<0.05, *versus* control cells. (D) Membrane SDC-4 expression was analyzed by western blot using SDC-4 antibodies after membrane fractionation for cells transfected with SDC4WT-GFP (SDC4WT) or SDC-4 constructs (S179A, L188QQ or A198del). Specificity of SDC-4 antibodies was checked using SDC4-siRNA transfected cells (siRNA SDC-4) and siRNA negative control (SNC). (E) HUV-EC-C transfected with GFP plasmid (control) or SDC4WT-GFP (SDC4WT) were incubated with anti-β1 integrin antibodies or isotype control (red fluorescence) and analyzed under confocal microscopy (×400). Scale bars: 10 µm. The EGFP fluorescence indicates that SDC-4 is localized at the cell membrane. The immunostaining of β1 integrin was used a specific membrane cell marker. (F) Cells were transfected with GFP plasmid (control) or with SDC4WT-GFP (SDC4WT). Membrane heparan sulfate (HS) chain expression of GFP-positive cells was quantified by flow cytometry with specific anti-HS antibodies without cell permeabilization (red histogram) or with isotype control (black histogram).

We addressed the potential role of SDC-4 in regulating the biological effects of RANTES/CCL5 by transfecting HUV-EC-C endothelial cells, which express SDC-4 endogenously, with Green Fluorescent Protein-tagged wild-type (SDC4WT-GFP) or with GFP-tagged SDC-4 constructs mutated at three key sites (Fig. 1A). In the first construct (SDC4S179A-GFP), the amino acid residue Ser located in the C1 domain was substituted by an Alanine. Phosphorylation of Ser 179 (Ser 183 in rat) in the intracellular domain of SDC-4 has been shown to regulate protein interactions, such as PKCα association (Horowitz and Simons, 1998; Finsen et al., 2011). In the second construct (SDC4L188QQ-GFP, PIP_2_^−^), the three consecutive residues Y^188^KK in the cytoplasmic tail of SDC-4 were mutated to LQQ, a mutation that affects the PIP_2_ affinity of the cytoplasmic tail (Horowitz et al., 2002). In the third construct (SDC4A198del-GFP, PDZ^−^), the COOH-terminal residue (Ala^198^) was deleted, leading to a deficient PDZ-dependent protein binding of SDC-4 (Horowitz et al., 2002) (Fig. 1A). We first verified that the EGFP tag present in our construct in intracellular C terminus did not alter on its own the functionality of SDC-4. RANTES/CCL5-induced chemotaxis on HUV-EC-C cells is similar in cells transfected with plasmids encoding for SDC-4 without any tag, or for SDC-4 with a CFP tag in N-terminal position, or for SDC-4 with Myc-His tag at C-terminal position, or for SDC-4 with GFP at C-terminus (data not shown). To measure the expression level of the transfected constructs and to locate the distribution of the GFP-SDC-4 constructs, we have carried out flow cytometry and immunofluorescence experiments (Fig. 1B). As a negative control, cells were transfected with the vector encoding for GFP alone (control). Flow cytometry analyses were carried out on non-permeabilized cells in order to detect by the use of red-labelled anti-SDC-4 antibodies, the SDC-4 present at the cell surface. For each SDC-4 constructs, the transfection efficiency, assessed by EGFP fluorescence intensity, ranges from 35 to 46% and was similar among the different constructions. The SDC-4 expression quantified by flow cytometry at the membrane of non-permeabilized GFP positive cells was similar whatever the SDC-4 constructs overexpressed by the cells (Fig. 1C). The relative expression of each SDC-4 (SDC4WT-GFP, SDC4S179A-GFP, SDC4L188QQ-GFP or SDC4A198del-GFP) construct to the SDC4-WT was also assessed in the membrane fraction after cell fractionation, by Western-blot (Fig. 1D). The specificity of anti-SDC-4 antibody was verified by the reduced detection of SDC-4 molecules in cells transfected with a specific SDC-4 small interfering RNA (siRNA). Whatever the SDC-4 variant, the protein amounts of SDC-4 detected in the membrane fraction were almost similar (Fig. 1D). The SDC-4 localization at the cell membrane was also evidenced by confocal microscopy (Fig. 1E). In these experiments, the membrane is underlined by the staining of β1 integrin chains, a typical membrane cell marker (Fig. 1E). Confocal analysis demonstrated that all SDC-4 constructs, including SDC4L188QQ-GFP, encode proteins expressed at the endothelial cell surface (data not shown). It is to note that overexpressed SDC-4 molecules also aggregate into the cells in all conditions. The degree of sulfatation of heparan sulfate chains is essential for the binding of RANTES/CCL5. To avoid any experimental bias, we next addressed the question whether SDC-4 construct overexpression could lead to a saturation of heparan sulfate chain biosynthesis enzymes, and therefore to lower sulfated heparan sulfate chains. The degree of sulfatation of syndecan-4 glycosaminoglycan chains is essential for the binding of RANTES/CCL5 (Gandhi and Mancera, 2008). Heparan sulfate chains present at the cell surface were increased by the SDC-4 overexpression as assessed by flow cytometry using specific anti-heparan sulfate antibodies (Fig. 1F). Moreover, the levels of heparan sulfate chains were similar whatever the SDC-4 variant overexpressed (data not shown). As assessed by real-time RT-PCR, the levels of mRNA encoding for EXT1 and EXT2, which are involved in the first step of heparan sulfate chain biosynthesis, were unaffected by SDC-4 overexpression (data not shown).

**Fig. 2.**
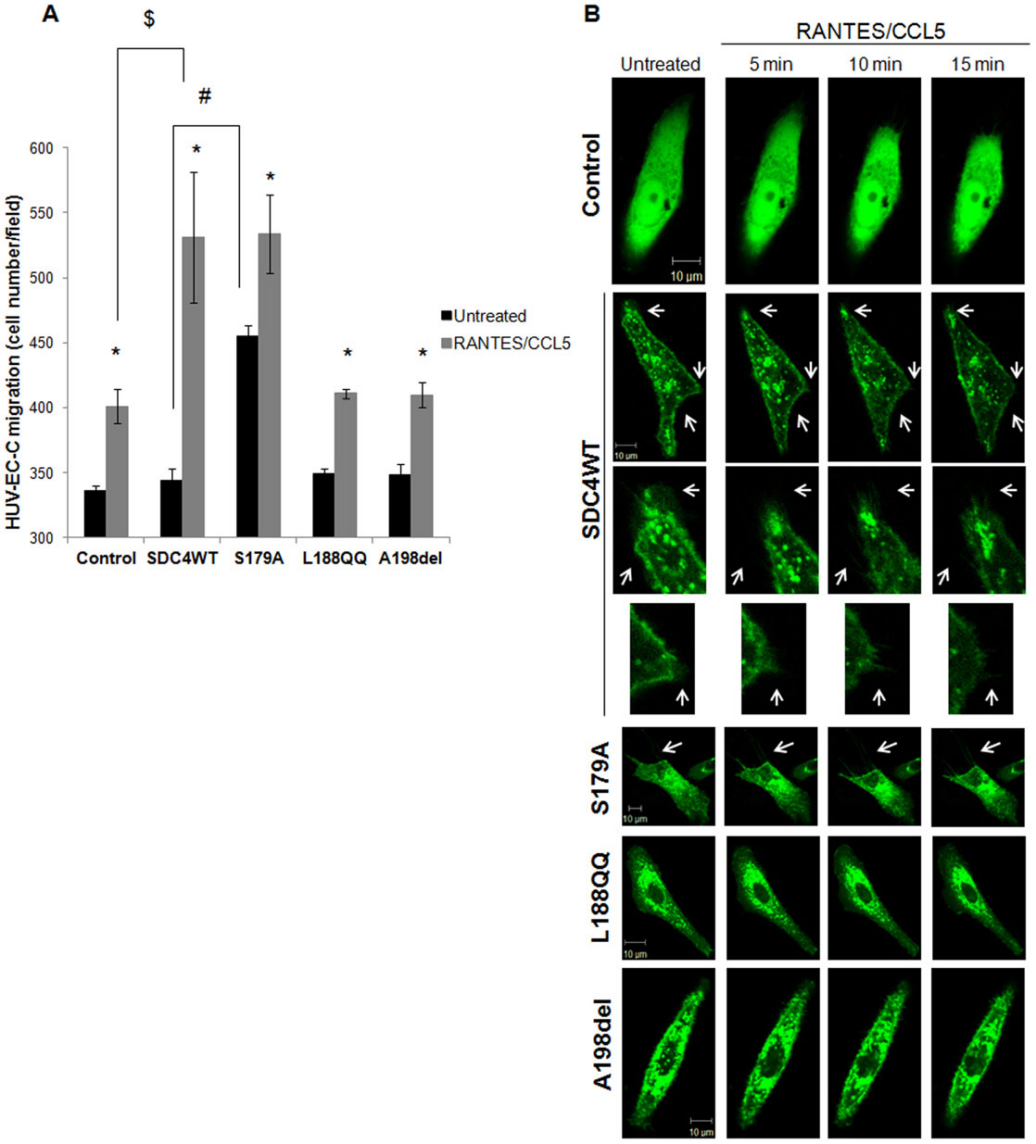
Intracellular domains of syndecan-4 are involved in RANTES/CCL5-mediated HUV-EC-C migration. (A) HUV-EC-C transfected with GFP plasmid (control) or with SDC4WT-GFP (SDC4WT) or with SDC-4 constructs (S179A, L188QQ, or A198del) were stimulated or not by 3 nM RANTES/CCL5 and cell migration was assayed by a transwell chamber model. Results are indicated as cell number/field (mean ± SEM). The vertical axis ranges from 300 to 600 cells/field. * P<0.05, RANTES/CCL5 *versus* control; # P<0.05, S179A *versus* SDC4WT (in the absence of RANTES/CCL5); $ SDC4WT *versus* control (in the presence of RANTES/CCL5). (B) The morphology of transfected HUV-EC-C was analyzed by live confocal microscopy upon RANTES/CCL5 stimulation for 15 minutes. (×400). Scale bars: 10 µm. Membrane protrusions were shown by white arrows.

Migration rate in response to RANTES/CCL5 treatment was measured by modified Boyden chamber experiments. As shown in Fig. 2A, in the absence of stimulation by the chemokine, SDC4WT-GFP, SDC4L188QQ-GFP, SDC4A198del-GFP-transfected endothelial cell migration was unchanged as compared to vector-transfected ones (control). By contrast, SDC4S179A-GFP-transfected cell migration was increased by 32±3% as compared to SDC4WT-transfected cell migration. RANTES/CCL5 increased vector-transfected cell migration by 19±3% as compared to vector-transfected cell migration towards medium alone (401±13 cells/field *versus* 336±4 cells/field, n = 3, P<0.05). The chemotactic effect of RANTES/CCL5 was higher in SDC4WT-GFP-transfected cells as it increased SDC4WT-GFP-transfected cell migration by 55±9% as compared to SDC4WT-GFP-transfected cell migration towards medium alone (531±50 cells/field *versus* 344±10 cells/field, n = 3, P<0.05). RANTES/CCL5 treatment led to a similar induction of cell migration after over-expression of SDC4S179A-GFP (17±4%) or SDC4L188QQ-GFP (PIP_2_^−^) (18±1%) or SDC4A198del-GFP (PDZ^−^) (18±3%). Indeed, cell number/field was 455±8 in unstimulated SDC4S179A-GFP cells *versus* 534±30 in SDC4S179A-GFP cells stimulated by RANTES/CCL5, 349±4 in unstimulated SDC4L188QQ-GFP (PIP_2_^−^) cells *versus* 411±4 in SDC4L188QQ-GFP cells stimulated by RANTES/CCL5 or 349±9 in unstimulated SDC4A198del-GFP (PDZ^−^) cells *versus* 410±10 in SDC4A198del-GFP cells stimulated by RANTES/CCL5 (Fig. 2A). These results were confirmed by a migration wound healing assay and an invasion transwell assay (data not shown).

Cell migration involves formation of a leading edge in the direction of migration and adhesion points from which tension is generated to move the cell body forward. Disassembly of adhesion points occurs at the back of the cell, a region known as the trailing edge. In order to analyze the morphology of the SDC-4 transfected-cells, live fluorescent microscopy was carried out. This technique enables the visualization of only one cell per observation field and was repeated six times. Upon RANTES/CCL5 induction, SDC4-GFP molecules preferentially localize at the leading lamella and along the trailing edge of migratory SDC4WT-GFP-transfected cells (Fig. 2B; supplementary material Movie 1). SDC4S179A-GFP-transfected cells display morphology similar to the SDC4WT-transfected cells (Fig. 2B). Interestingly, endothelial cells expressing SDC4L188QQ-GFP (PIP_2_^−^) or SDC4A198del-GFP (PDZ^−^), whereas also forming lamellipodia, failed to polarize by forming leading and trailing edge when compared with cells expressing SDC4WT-GFP (Fig. 2B). The reduction of cell area may be associated with cell migration properties. The area of cells transfected with SDC4WT-GFP or SDC4S179A-GFP was reduced upon RANTES/CCL5 treatment by 11±4% and 13±3% respectively (n = 6, P<0.05). In contrast, cells transfected either with SDC4L188QQ-GFP or with SDC4A198del-GFP exhibit similar area after RANTES/CCL5 treatment as compared to cells expressing SDC4WT-GFP (0±2% and 3±2% respectively, n = 6).

**Fig. 3.**
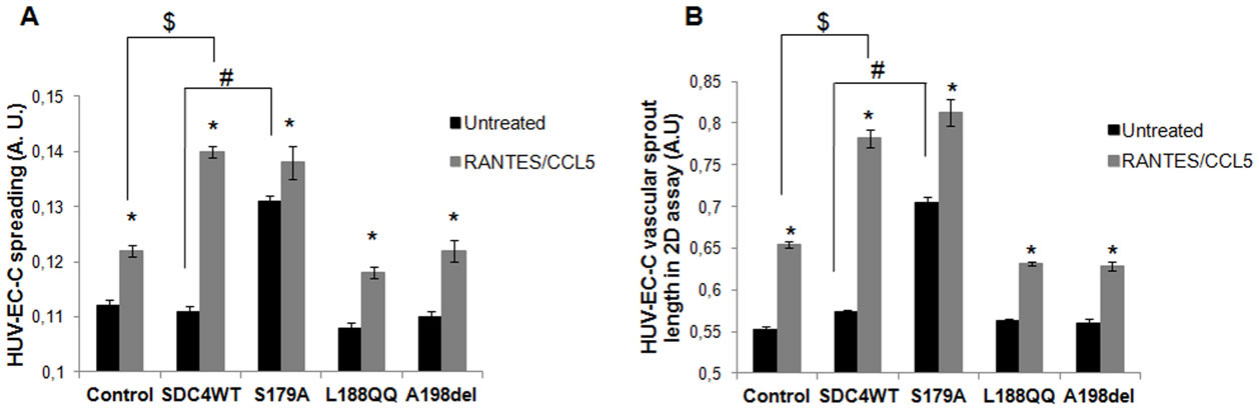
Intracellular domains of syndecan-4 are involved in RANTES/CCL5-mediated HUV-EC-C spreading and vascular tube formation. HUV-EC-C transfected with GFP plasmid (control), SDC4WT-GFP (SDC4WT) or mutated SDC-4 constructs (S179A, L188QQ, A198del) were stimulated or not by 3 nM RANTES/CCL5 and assayed for cell spreading on fibronectin (A) or vascular tube formation in Matrigel (B). Results are expressed as area (A) or as length of vascular sprout (B) (mean ± SEM) expressed in arbitrary units (A.U.). The vertical axis ranges from (A) 0.1 to 0.15 or from (B) 0.5 to 0.85 A.U. * P<0.05, RANTES/CCL5 *versus* control; # P<0.05, S179A *versus* SDC4 (in the absence of RANTES/CCL5); $ P<0.05 SDC4WT *versus* control (in the presence of RANTES/CCL5).

Cell spreading participates at the highly integrated multistep process leading to cell migration. We then assayed RANTES/CCL5-induced spreading of endothelial cells transfected with SDC4WT-GFP or with SDC-4 mutants. In the absence of any RANTES/CCL5 stimulation, the spreading of endothelial cells expressing SDC4S179A-GFP was increased by about 18% as compared to SDC4WT-GFP expressing cells (0.131±0.001 *versus* 0.111±0.001, n = 3, P<0.05) (Fig. 3A). The spreading of endothelial cells expressing either wild-type SDC-4 or SDC-4 deleted in the PIP_2_ or PDZ regions was unchanged as compared to vector-transfected ones (control) (Fig. 3A). RANTES/CCL5 increased the spreading of SDC4WT-GFP-transfected cell by 26±1% as compared to untreated ones (0.140±0.001 *versus* 0.111±0.001, n = 3, P<0.05). The spreading induction in response to RANTES/CCL5 treatment of cells expressing either SDC4S179A-GFP (5±2%) or SDC4L188QQ-GFP (9±1%) or SDC4A198del-GFP (11±1%) were similar to the vector-transfected cells (control, 9±1%) (Fig. 3A).

As RANTES/CCL5 has been demonstrated to exert pro-angiogenic effects (Suffee et al., 2012), angiogenesis assay was tested on SDC-4 construct-transfected cells upon chemokine stimulation. In the absence of stimulation by RANTES/CCL5, the formation of vascular sprout, as assessed by vascular sprout length, was increased in cells expressing SDC4S179A-GFP by 28±1% increase as compared to vector-transfected cells (control) but was unchanged when cells were transfected with SDC4WT-GFP, SDC4L188QQ-GFP or SDC4A198del-GFP constructs. The endothelial vascular sprout length upon RANTES/CCL5 stimulation of cells expressing SDC4WT-GFP was increased as compared to unstimulated ones (0.782±0.011 *versus* 0.574±0.002, corresponding to an increase of 36±1%, n = 3, P<0.05). RANTES/CCL5 increased to a lesser extent the endothelial vascular sprout length of cells expressing SDC4S179A-GFP (0.813±0.016 *versus* 0.705±0.007, corresponding to an increase of 15±2%, n = 3, P<0.05) orSDC4L188QQ-GFP (0.632±0.002 *versus* 0.563±0.003, corresponding to an increase of 12±1%, n = 3, P<0.05) or SDC4A198del-GFP (0.629±0.005 *versus* 0.560±0.006, corresponding to an increase of 12±1%, n = 3, P<0.05) as compared to the unstimulated respective cells (Fig. 3B).

Altogether, biological effects induced by the chemokine RANTES/CCL5 were largely decreased when cells over-expressed SDC4S179A, SDC4L188QQ or SDC4A198del.

### RANTES/CCL5 biological effects depend on the syndecan-4/PKCα signaling pathway

**Fig. 4.**
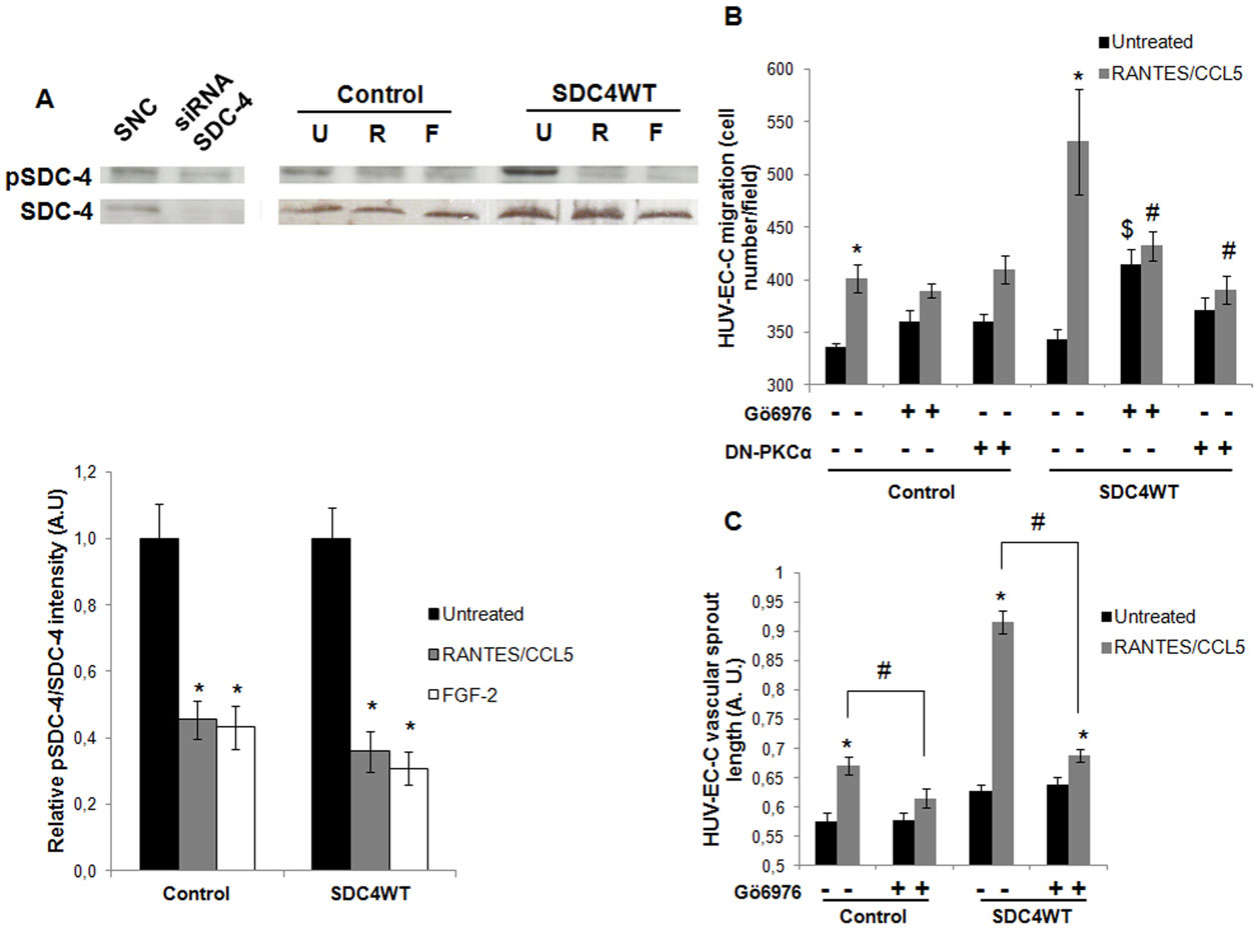
PKCα mediates RANTES/CCL5-induced endothelial cell migration and vascular tube formation via syndecan-4. (A) Specificity of SDC-4 and pSDC-4 antibodies was checked using siRNA-negative control (SNC) or siRNA-SDC-4 (siRNA SDC-4) transfected cells by western blot analysis. HUV-EC-C transfected with GFP plasmid (control) or with SDC4WT-GFP (SDC4WT) were stimulated or not (U) by 3 nM RANTES/CCL5 (R) or 20 ng/ml FGF-2 (F) and SDC-4 phosphorylation at Ser179 was evaluated by western blot. Upper panel, representative Western blot analysis. Lower panel, densitometry quantification of three independent experiments. pSDC-4 band intensity was normalized to SDC-4 one. Results of relative densitometry intensities (mean ± SEM) are expressed in arbitrary units (A.U.). * P<0.05, RANTES/CCL5 or FGF-2 *versus* unstimulated cells. (B,C) HUV-EC-C transfected with GFP plasmid (control) or with SDC4WT-GFP (SDC4WT) were either co-transfected with a dominant negative PKCα plasmid (DN-PKCα). They were pre-incubated or not with Gö6976, a PKCα inhibitor, and treated or not with 3 nM RANTES/CCL5. They were then assayed for cell migration in a transwell chamber model (B) or for vascular tube formation in Matrigel (C). (B) Results are expressed as mean ± SEM of migrated cell number/field. Vertical axis ranges from 300 to 600 cells/field. (C) Results of vascular sprout length are presented as mean ± SEM expressed in arbitrary units (A.U.). Vertical axis ranges from 0.5 to 1 A.U. * P<0.05, RANTES/CCL5 *versus* untreated cells; $ P<0.05 cells treated with Gö6976 *versus* cells in the absence of PKCα inhibitor (in the absence of RANTES/CCL5); # P<0.05 cells preincubated with Gö6976 or with the dominant negative PKCα plasmid *versus* cells in the absence of PKCα inhibitor (in the presence of RANTES/CCL5).

A demonstrated signaling role of syndecan-4 is the modulation of FGF-2-stimulated PIP_2_-dependent PKCα activity. We therefore addressed the question whether PKCα was activated when transfected endothelial cells are stimulated by RANTES/CCL5. Prior studies have established that dephosphorylation of Ser-179 in SDC-4 cytoplasmic domain is required for PKCα activation (Horowitz and Simons, 1998). RANTES/CCL5 treatment induced Ser-179 dephosphorylation in endothelial cells transfected with SDC4WT-GFP, in a way similar to FGF-2 used as positive control (Fig. 4A). The less intense expression of SDC-4 and pSDC-4 revealed by western blot using anti-SDC-4 and anti-pSDC-4 antibodies in cells transfected with siRNA SDC-4, attested the specificity of these antibodies (Fig. 4A). In subsequent studies, the involvement of PKCα in RANTES/CCL5-induced biological effects was tested either by incubating cells with Gö6976, or by the use of transfected dominant negative plasmid. Gö6976 is a potent and selective PKCα inhibitor (IC50 = 2.3 nmol/L for PKCα), but does not inhibit the activity of PKCδ, -ε, or -ζ (Chen et al., 2014). Upon Gö6976 cell treatment, endothelial cell migration and vascular tube formation induced by RANTES/CCL5 were largely decreased in SDC4WT-transfected cells as compared to SDC4WT-transfected cells in the absence of inhibitor (Fig. 4B,C). These data were confirmed and even more pronounced after PKCα inhibition by the transfection with a dominant negative PKCα plasmid. Strikingly, in the absence of RANTES/CCL5 stimulation, endothelial cell migration was increased upon Gö6976 cell treatment, conversely to HUV-EC-C vascular sprout length.

**Fig. 5.**
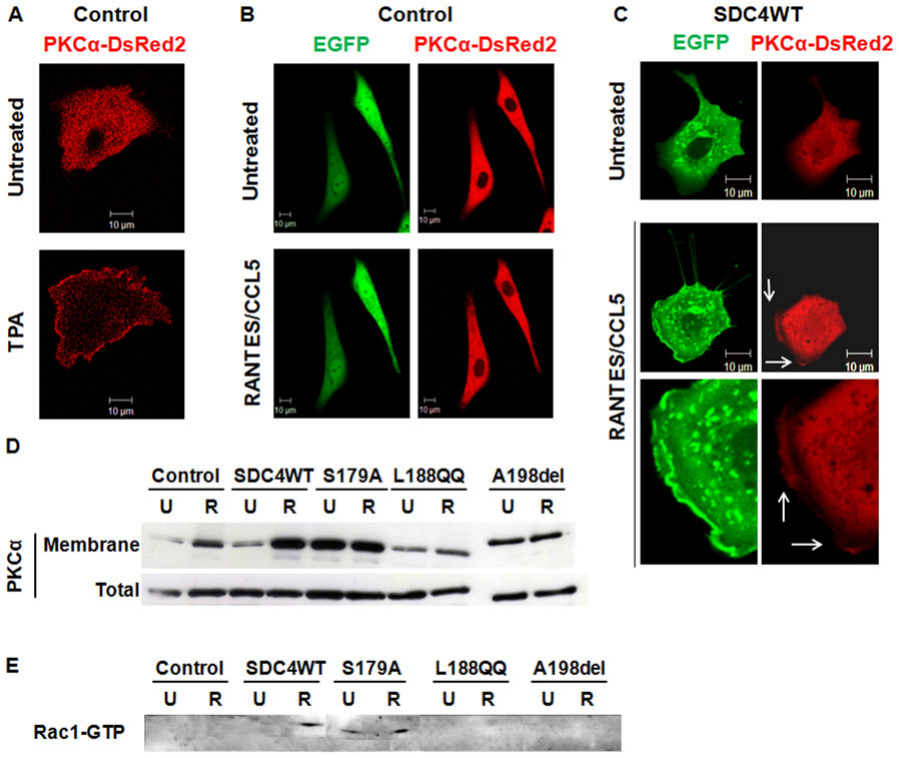
RANTES/CCL5 induced co-localization of SDC-4 and PKCα at the cell membrane and Rac1 activation. (A–C) HUV-EC-C were co-transfected with PKCα-DsRed2 plasmid and with either GFP plasmid (control, panels A and B) or GFP-SDC4WT (SDC4WT, panel C). They were incubated or not with (A) 0.5 µM TPA or with (B,C) 3 nM RANTES/CCL5 for 15 minutes and analyzed under live confocal microscopy. Membrane localization of SDC-4 (green) and PKCα (red) was indicated with white arrows. (×400). (D) HUV-EC-C transfected with GFP plasmid (control) or with SDC4WT-GFP (SDC4WT) or with mutated SDC-4 constructs (S179A, L188QQ, A198del) were stimulated or not (U) by 3 nM RANTES/CCL5 (R). After cell fractionation, the amount of PKCα in membrane of total fraction was evaluated by western blot. (E) HUV-EC-C transfected with GFP plasmid (control) or with SDC4WT-GFP (SDC4WT) or with mutated SDC-4 constructs (S179A, L188QQ, A198del) were stimulated or not (U) by 3 nM RANTES/CCL5 (R). Rac1-GTP activity was determined by pull down assay and analyzed using specific Rac1-GTP antibodies by western blot. Scale bars: 10 µm.

Finally, endothelial cells were co-transfected with SDC4WT-GFP or the SDC4-GFP mutants and with a Ds-red PKCα and their localization was visualized with a confocal microscope. The low red signal associated with PKCα and the high green signal due to the intracellular accumulation of SDC4WT-GFP do not allow the precise quantification of co-localized signals. As control, 12-O-tetradecanoylphorbol-13-acetate (TPA) induces the translocation of Ds-red PKCα at the cell membrane (Fig. 5A). RANTES/CCL5 treatment of co-transfected endothelial cells exerts no effect on cells transfected with empty plasmid but induces membrane localization of Ds-Red PKCα and SDC4WT-GFP, especially visible in areas where membrane protrusions appear distinctly, suggesting the membrane translocation of PKCα leading to its activation (Fig. 5C; supplementary material Movie 2). The effect of RANTES/CCL5 on PKCα translocation to the membrane was assessed by western blot after fractionation of cells overexpressing SDC4WT-GFP, SDC4S179A-GFP, SDC4L188QQ-GFP or SDC4A198del-GFP. RANTES/CCL5 induced PKCα translocation to the membrane in cells transfected with empty plasmid, and even more in cells overexpressing SDC-4. The SDC4S179A-GFP overexpression leads to an increased total expression of PKCα associated with a high level of PKCα at the cell membrane, unchanged by the stimulation with RANTES/CCL5. This result was confirmed by confocal microscopy (supplementary material Movie 3). Whereas cells transfected with SDC4A198del-GFP exhibit intermediate amounts of PKCα expressed at the cell membrane, the SDC4L188QQ-GFP variant inhibits the PKCα translocation (Fig. 5D; supplementary material Movie 4).

Similarly, the effect of RANTES/CCL5 on Rac1 activation was assessed by a pull-down assay in cells overexpressing SDC4WT-GFP, SDC4S179A-GFP, SDC4L188QQ-GFP or SDC4A198del-GFP. RANTES/CCL5 induced Rac1 activation only in cells transfected with SDC4WT-GFP or SDC4S179A-GFP. The cell transfection with SDC4S179A-GFP induces Rac1-GTP activation in the absence of RANTES/CCL5 (Fig. 5E).

## DISCUSSION

We have recently demonstrated the proangiogenic role of the chemokine RANTES/CCL5 by the use of *in vitro* and *in vivo* experimental approaches (Suffee et al., 2012). RANTES/CCL5-induced proangiogenic effects depend both on CCR1, its G-protein coupled receptor, and also on glycosaminoglycans carrying by membrane proteoglycans belonging to the syndecan family, namely SDC-1 and -4. It was recently demonstrated that PGE_2_-induced ERK activation in endothelial cells and PGE_2_-induced angiogenesis are driven by SDC-4-dependent PKC activation (Corti et al., 2013). Our working hypothesis was that syndecan-4 molecules participate to RANTES/CCL5 signalling, leading to biological effects in endothelial cells. For that purpose, syndecan-4 constructs were established in the intracellular syndecan-4 domain. A Ser-to-Ala mutation in the C1 SDC-4 intracellular domain was introduced at position 179 (S183 in rat) and would have been expected to favor PKC activation (Horowitz and Simons, 1998; Murakami et al., 2002). In the second construct, the three consecutive residues Y^188^KK in the V domain were mutated to LQQ. This mutant has been described to have a reduced affinity to PIP_2_, leading to an inhibition of its PIP_2_-mediated PKC activation (Horowitz et al., 2002). The third construct has a deletion of A^198^, which abolished PDZ-dependent binding of syndecan-4 (Horowitz et al., 2002). The PDZ protein interaction domain of SDC-4 (EFYA amino-acid sequence) is very important for the syndecan-4-induced signaling. Some studies showed that the mutation of this domain altered the cell migration induced by FGF-2 via syndecan-4 signaling (Gao et al., 2000; Horowitz et al., 2002; Tkachenko et al., 2006). It was shown that the abolition of PDZ-binding in the SDC-4 intracellular domain failed to activates PKCα which is necessary for the FGF-2 dependant migration (Horowitz et al., 2002). SDC-4 PDZ deficient mutant is unable to bind the PDZ protein synectin. This abolition failed to activate and localize Rac1 at proximity of the leading edge, which is essential for the initiation of cell migration (Tkachenko et al., 2006). Spreading, migration and vascular tube formation induced by RANTES/CCL5 were largely impaired in SDC4L188QQ- or SDC4A198del-transfected cells as compared to the mock-transfected ones, suggesting that the chemokine biological activities are dependent on PKC activation. The biological activities mentioned above were not affected by the mutants under basal conditions (in the absence of RANTES/CCL5). By contrast, the overexpression of SDC4-S179A mutant highly raised endothelial cell spreading, migration and tube formation without any chemokine stimulation and leads to a reduced magnitude of the RANTES/CCL5 effects, as compared to mock-transfected cells. Therefore, these data give evidence that SDC-4 is a co-receptor for the chemokine RANTES/CCL5 by activating signaling through its own intracellular domains. Furthermore, RANTES/CCL5 dependence on the SDC-4/PKCα signaling pathway is demonstrated by a number of observations. First, RANTES/CCL5 biological activities are largely reduced when endothelial cells are incubated with a specific PKCα inhibitor or co-transfected with a dominant negative PKCα. Second, RANTES/CCL5 treatment of endothelial cells leads to the dephosphorylation of the Ser-179 site of SDC-4 cytoplasmic tail. This amino acid residue (S183 in rat) is crucial for PIP_2_-mediated PKCα binding to SDC-4 leading to PKCα activation. Third, western blotting analysis demonstrates that RANTES/CCL5 induced membrane translocation for SDC4WT-GFP transfected cells but not in SDC4L188QQ-GFP or SDC4A198del-GFP-transfected ones and confocal microscopy analysis demonstrates that RANTES/CCL5 induced the membrane localization of DsRed-Tagged PKCα and SDC4-GFP in SDC4WT-GFP tranfected endothelial cells. These data are consistent with those previously published whereby the SDC-4 interacts with PIP_2_ which allow the activation of PKCα (Oh et al., 1997; Oh et al., 1998). Regulation of Rho family GTPases may also lie downstream of PKCα (Bass et al., 2007; Bass et al., 2008; Dovas et al., 2006). Previous studies indicated that SDC-4 orchestrates the polarization of active Rac1 in the presence of chemotactic signals such as FGF-2 and that SDC-4 induces Rac1-dependent cell migration in a manner that requires both its PDZ-binding domain and PKCα (Tkachenko et al., 2006; Bass et al., 2007). Elfenbein et al. have also demonstrated that Rac1 activation downstream of SDC-4 is mediated by RhoG activation pathway (Elfenbein et al., 2009). Rac1 activation has been shown to be critical for both CCR1- and CCR5-triggered signaling cascades mediating RANTES/CCL5-induced reorganization of the actin cytoskeleton (Di Marzio et al., 2005). It has been shown that RANTES/CCL5 mediated T-cell activation and chemotaxis requires Rho GTPase activity (Clissi et al., 2000). We therefore hypothesize that SDC-4 may probably participate to RANTES/CCL5 biological activities by activating members of the Rho family of small GTPases, and we demonstrated by pull down assay that RANTES/CCL5 induced Rac1 activation for SDC4WT-GFP transfected endothelial cells but not in SDC4L188QQ-GFP or SDC4A198del-GFP-transfected ones.

Our data also highlight that SDC-4 participates to RANTES/CCL5-mediated biological effects, such as cell migration or vascular tube formation in a PDZ domain-dependent manner since chemokine activities were impaired in SDC4A198del-GFP-transfected endothelial cells. PDZ domains are protein interaction modules that regulate targeting and trafficking of cell surface proteins. It has been previously demonstrated that SDC-4 promotes endothelial cell migration in response to ligand binding by activating Rac1 and localizing it to the leading edge and that these processes are dependent on its PDZ-binding domain interaction with synectin, a small intracellular scaffold protein (Tkachenko et al., 2006; Grootjans et al., 1997). FGF-2-induced Rac1 activation depends on the suppression of RhoG by a SDC4-synectin-RhoGDI1 complex and activation via PKCα (Elfenbein et al., 2009). Syntenin, the first-described syndecan-binding partner, binds also to SDC-4, leading to a regulation of integrin recycling (Morgan et al., 2013). The identification and the precise role of PDZ proteins interacting with SDC-4 in RANTES/CCL5 activities are actually unknown.

In summary, our data demonstrate that SDC-4 is a typical co-receptor for the chemokine RANTES/CCL5 and that the interaction of both partners leads to activation of PKCα through the intracellular domain of SDC-4. Regarding the multiple role of RANTES/CCL5 in various pathologies, including cancer, viral diseases and inflammation, deciphering the mechanism by which RANTES/CCL5 exerts its biological activities is a preliminary step to develop new therapeutic strategy, for example by targeting the binding of the chemokine to its proteoglycan receptor.

## MATERIALS AND METHODS

### Antibodies and reagents

RANTES/CCL5 was synthesized by L. Martin and C. Vita (CEA Saclay, Gif-sur-Yvette, France) as previously described (Charni et al., 2009) and used at 3 nM. Fibronectin (100 µg/ml) and Matrigel (320 µg/ml) were from BD Biosciences Pharmingen (Le Pont de Claix, France). Mayer's Hemalun (a nucleus marker) was from Roth (Lauterbourg, France). Crystal Violet (0.1%), TPA (0.5 µM, 12-O-tetradecanoylphorbol-13-acetate), FGF-2 (20 ng/ml, Fibroblast Growth Factor-basic) and PKCα/β1 inhibitor Gö6976 (1 µM) were from Sigma–Aldrich (Saint-Quentin Fallavier, France). Antibodies were used at a 10 µg/ml concentration: primary antibodies mouse IgG2a anti-human SDC-4 (5G9) and mouse IgG1 anti-human integrin β1 were from Santa Cruz Biotechnology (Tebu Bio, Le Perray-en-Yvelines, France), mouse IgM anti-human heparan sulfate (F58-10E4) was from Seikagaku Biobusiness Corporation (Tokyo, Japan). Isotype controls, mouse IgG1, mouse IgG2a and mouse IgM, were from BD Biosciences Pharmingen. Secondary antibodies Alexa Fluor 555-goat anti-mouse IgG and Alexa Fluor 647-goat anti-mouse IgG were from Invitrogen (Life Technology), APC-rat anti-mouse IgM was from BD Biosciences Pharmigen. For Western blotting analysis, primary antibodies goat IgG anti-human pSDC-4 (Ser 179), rabbit IgG anti-human SDC-4 (H140) were from Santa Cruz Biotechnology (Tebu Bio) and mouse IgG2b anti-human PKCα was from BD Biosciences Pharmigen; isotype controls goat IgG and mouse IgG were from Santa Cruz Biotechnology (Tebu Bio) and rabbit IgG was from R&D Systems (Lille, France); secondary antibodies HRP-donkey anti-goat IgG was from Santa Cruz Biotechnology (Tebu Bio), HRP-donkey anti-mouse IgG and HRP-donkey anti-rabbit IgG was from Jackson ImmunoResearch (Immunotech S.A.S, Marseille, France).

### cDNA contructs

Syndecan-4 cDNA (OriGene, CliniSciences, Nanterre, France) was inserted in pEGFP-N3 plasmid (Clontech, Ozyme, Saint-Germain-en-Laye, France) between EcoRI and KpnI restriction sites. Specific mutations for SDC-4 sequence were performed with QuikChange II Site-Directed Mutagenesis Kit (Stratagene, Agilent Technologies, Les Ulis, France). Briefly, pEGFP-N3-SDC-4 (50 ng) and primers (125 ng of upper primer and 125 ng of lower primer) for each mutation were mixed with reaction buffer (10 mM Tris-HCl, 1 mM EDTA, pH 7.5), 1 µL dNTPmix and 2.5 U PfuUltra HF DNA polymerase, and PCR was performed (1 step 30 seconds at 95°C, 18 stages of 3 steps (30 seconds at 95°C, 1 minute at 55°C, 6 minutes at 68°C). The amplified PCR products were then digested by DpnI for 1 hour.

pEGFP-N3 (Control), pEGFP-N3-SDC-4 wild-type (SDC4WT), pEGFP-N3-SDC-4-S179A (S179A), pEGFP-N3-SDC-4-L188QQ (L188QQ), pEGFP-N3-SDC-4-A198del (A198del) plasmids were transformed in XL1-Blue Supercompetent cells (*E. coli*, Stratagene) according to manufacturer's instructions and sequences were checked by Beckman Coulter Genomics (Takeley, United Kingdom).

PKCα-DsRed2 and Dominant-Negatif PKCα-K368M-DsRed2 plasmids were constructed by Pr. N. Saito team (Masukawa et al., 2006).

Plasmids amplification and purification were performed using miniprep and maxiprep kits (Qiagen, Courtaboeuf, France; Masukawa et al., 2006) according to manufacturer's protocols.

### Cell culture, transfection and transduction

Human umbilical vein endothelial cells (HUV-EC-C, no. CRL-1730, ATCC) were cultured as previously described (Suffee et al., 2012).

HUV-EC-C were harvested and 10^6^ cells were incubated with 5 µg of plasmid in 100 µl Amaxa cell line nucleofector solution V (Lonza). Cells were transfected using protocol V-001 of AMAXA nucleofector device II (Lonza). Transfected cells were cultured at 10^6^ cells/ml in ECBM2 containing 12% of fetal calf serum. After 8 hours, dead cells were removed and fresh medium was added. For all experiments, cells were used 24 hours after transfection.

### Flow cytometry

The cell transfection efficiency with the various plasmids was analyzed 24 hours after transfection by flow cytometry by the measure of EGFP fluorescence intensity. SDC-4 overexpression at endothelial cell membrane was assessed by the use of specific antibodies directed against SDC-4 extracellular domain or with isotype controls revealed by Alexa Fluor 647-goat anti mouse IgG as secondary antibodies. Heparan sulfate chain expression at endothelial cell membrane was assessed using specific anti-heparan sulfate antibodies or isotype controls revealed by APC-rat anti-mouse IgM as secondary antibodies.

SDC-4 expression was analyzed by the detection of EGFP fluorescence with a confocal microscope 24 hours after transfection. The membrane localization of SDC-4 was evidenced by a merged fluorescence of EGFP and integrin β1 immunostaining, a membrane marker, with specific antibodies and Alexa Fluor 555-goat anti mouse IgG as secondary antibodies.

### Cell spreading

Transfected cells, incubated for 2 hours with or without RANTES/CCL5 were stained with Alexa Fluor 568-phalloidin (1:200, Invitrogen) and observed with a fluorescence microscope (Zeiss, AXIOPHOT, N°/MicMac, Le Pecq, France) as previously described (Charni et al., 2009). Ten fields of stained cells were photographed and cell areas were evaluated on 40 cells with Scion Imager (Scion Image Software and National Institutes of Health, Release Beta 3b Software).

### Cell migration

Cell migration was analyzed in Boyden transwell migration chambers (Beckton Dickinson, Le Pont de Claix, France) as previously described (Sutton et al., 2007). Inserts of Boyden cell migration chamber were coated with fibronectin and 5×10^4^ transfected or co-transfected cells pre-treated or not 2 hours with Gö6976, a specific PKCα and PKCβ1 inhibitor, were incubated 24 hours at 37°C. In the lower chamber, medium supplemented or not with RANTES/CCL5 was added. After staining with Mayer's hemalum, cells were quantified.

### 2D-angiogenesis

For 2D angiogenesis assay, 1.5×10^4^ transfected cells were seeded on Matrigel-coated 8 wells Labtek for 24 hours with or without RANTES/CCL5 pre-incubated or not for 2 hours with Gö6976, a specific PKC inhibitor (Suffee et al., 2012). Cells were fixed, stained with Crystal Violet (Sigma–Aldrich) and photographed under phase contrast microscope (Olympus CK40, Rungis, France). The length of 30 vascular sprouts was evaluated using Scion Imager (Scion Imager Software).

### Live fluorescent microscopy

HUV-EC-C were co-transfected with pEGFP-N3, pEGFP-N3-SDC-4 wild-type or SDC-4 mutated plasmids and with PKCα-DsRed2 plasmid and seeded on a glass bottom dish (MatTek Corporation, Ashland, MA, USA). After 24 hours, co-transfected cells were incubated or not with RANTES/CCL5, and the localization of PKCα was monitored by confocal laser scanning fluorescence microscopy (model LSM 510 invert, Carl Zeiss, Jena, Germany). EGFP-SDC-4 was monitored at 488-nm argon excitation using a 510- to 535-nm band pass barrier filter. PKCα-DsRed2 was monitored at 543-nm HeNe1 excitation using a 590-nm-band pass barrier filter. DsRed2 and EGFP were monitored simultaneously using multitracking software which alternately detects each fluorescence by switching quickly between laser and filter system.

### Syndecan-4 phosphorylation

2×10^6^ HUV-EC-C transfected cells were cultured for 24 hours and incubated at 37°C for 15 minutes with or without 3 nM RANTES/CCL5 or 20 ng/ml FGF-2 and lysed in a buffer containing phosphate-buffered saline supplemented with 1% NP-40, 10 mM PMSF, 5 mM iodoacetamide, 25 mM *o*phenanthroline, 20 µg/ml aprotinin and 1 mM orthovanadate. Lysates were obtained by centrifugation at 10,000 × g for 15 minutes at 4°C and protein concentration was determined using the BCA protein assay kit (Pierce, Thermo Fisher Scientific, Brébières, France). 22 µg proteins were loaded on SDS-PAGE to reveal unphosphorylated SDC-4 on Ser-179, total SDC-4 using specific antibodies, purchased from Santa Cruz Biotechnology (Tebu Bio): anti-p-syndecan-4 (Ser179) at 0.5 µg/ml, anti-syndecan-4 (rabbit polyclonal IgG, clone H140) at 1 µg/ml. Revelation was performed using horseradish peroxidase-conjugated anti-goat IgG (at 0.2 µg/ml, Santa Cruz Biotechnology, Tebu Bio) or anti-rabbit IgG (at 0.16 µg/ml, Jackson Immuno Research).

### PKCα membrane translocation

2×10^6^ HUV-EC-C transfected cells were cultured for 24 hours and incubated at 37°C for 15 minutes with or without 3 nM RANTES/CCL5. Cell fractionation was performed using Subcellular Protein Fractionation Kit for Cultured Cells from Pierce according to manufacturer's protocol. Briefly, cells were harvested, wash with cold PBS and pellet was obtained by centrifugation at 500 × g for 5 minutes at 4°C. Cell pellet was gently mixed with CEB buffer for 10 min at 4°C and cytoplasmic fraction was collected by centrifugation at 500 × g for 5 minutes at 4°C. Cell pellet was gently mixed with MEB buffer for 15 minutes at 4°C and membrane fraction was collected by centrifugation at 3,000 × g for 5 minutes at 4°C. Protein concentration was determined using the BCA protein assay kit (Pierce, Thermo Fisher Scientific, Brébières, France). 10 µg proteins were loaded on SDS-PAGE to reveal PKCα using specific mouse IgG2b anti-human PKCα antibody purchased from BD Biosciences Pharmigen at 0.5 µg/ml. Revelation was performed using horseradish peroxidase-conjugated anti-mouse IgG at 0.16 µg/ml purchased from Jackson Immuno Research.

### Rac-1-GTP pull down

2×10^6^ HUV-EC-C transfected cells were cultured for 24 hours and incubated at 37°C for 15 minutes with or without 3 nM RANTES/CCL5. Quantity of Rac1-GTP (active form) was determined using Rac1 Activation Magnetic Beads Pulldown Assay from Merck Millipore (Millipore S.A.S, Guyancourt, France) according to manufacturer's protocol. Briefly, cells were washed 2 times with cold PBS and were lysed in MLB buffer (25 mM HEPES, 150 mM NaCl, 50 mM MgCl_2_, 5 mM EDTA, 1% Igepal CA-630, glycerol 10%, aprotinin 10 µg/ml, leupeptine 10 µg/ml, orthovanadate 1 mM, pH 7.5). Lysates were incubated with 10 µg of Pak-1 PBD magnetic beads and gently mixed for 45 minutes at 4°C (binding of Rac1-GTP to the beads). Beads containing active Rac1 were washed 3 times with MLB buffer to remove all inactive Rac1 and were loaded on SDS-PAGE to reveal Rac-1 using supplied specific mouse IgG2a anti-human Rac1 antibodies at 1 µg/ml. Revelation was performed using horseradish peroxidase-conjugated anti-mouse IgG at 0.16 µg/ml purchased from Jackson Immuno Research.

### Statistical analysis

Results are presented as mean ± SEM. Statistical significance was assessed by one-way analysis of variance (ANOVA) test performed with the Statview software (StatView 4.5 Abacus Concepts, Berkeley, CA, USA). A P value of <0.05 was used as the criterion of statistical significance.

## Supplementary Material

Supplementary Material
