# Supplementary material for: RANTES/CCL5 mediated-biological effects depend on the syndecan-4/PKCα signaling pathway
Source: Biol Open. 2014 Sep 26;3(10):995–1004. doi: 10.1242/bio.20148227 (PMC4197448; doi:10.1242/bio.20148227)
Supplement: Supplementary Material [file supp_bio.20148227_bio.20148227-s1.pdf]

Biology Open (2014) 000, 1–10 doi:10.1242/bio.20148227

Supplementary Material

Loïc Maillard et al. doi: 10.1242/bio.20148227

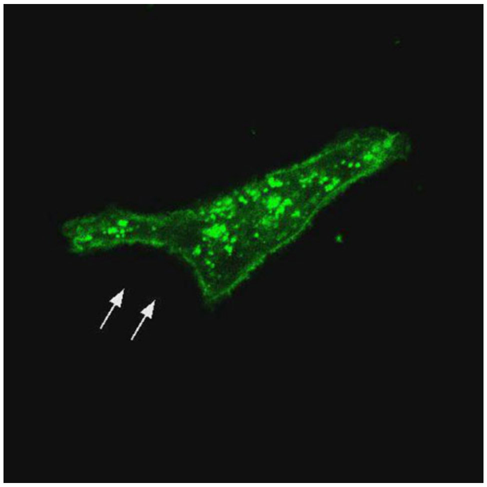

Movie 1. Morphology of SDC4WT-transfected HUV-EC-C. The morphology of SDC4WT-transfected HUV-EC-C was analyzed by live confocal microscopy upon RANTES/CCL5 stimulation for 15 minutes. Membrane protrusions were shown by white arrows. (×400).

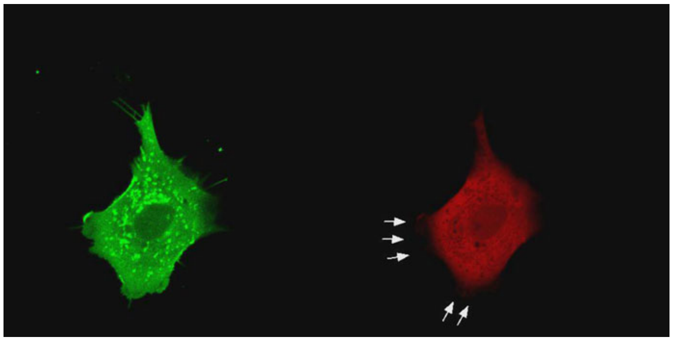

Movie 2. PKCα translocation of SDC4WT and PKCα -co-transfected HUV-EC-C. The PKCα translocation of PKCα-DsRed2 and GFP-SDC4WT co-transfected HUV-EC-C was analyzed by live confocal microscopy upon RANTES/CCL5 stimulation for 15 minutes. Membrane localization of PKCα (red) was indicated with white arrows. (×400).

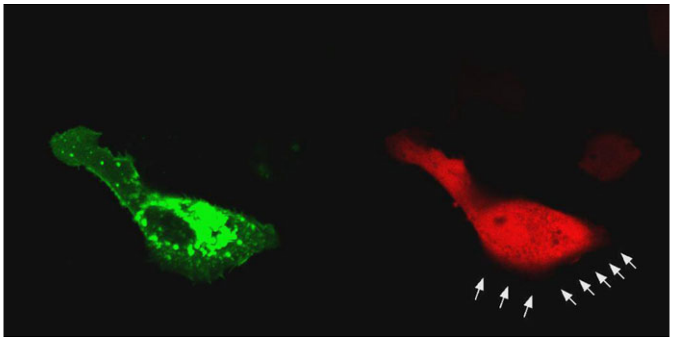

Movie 3. PKCα translocation of S179A and PKCα -co-transfected HUV-EC-C. The PKCα translocation of PKCα-DsRed2 and GFP-S179A-SDC4 co-transfected HUV-EC-C was analyzed by live confocal microscopy upon RANTES/CCL5 stimulation for 15 minutes. Membrane localization of PKCα (red) was indicated with white arrows. (×400).

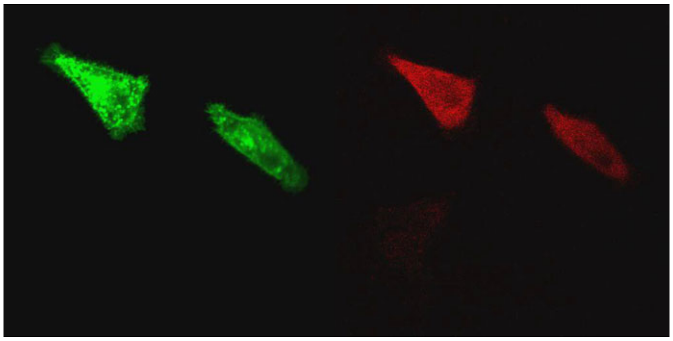

Movie 4. PKCα translocation of L188QQ and PKCα -co-transfected HUV-EC-C. The PKCα translocation of PKCα-DsRed2 and GFP-L188QQ-SDC4 co-transfected HUV-EC-C was analyzed by live confocal microscopy upon RANTES/CCL5 stimulation for 15 minutes. (×400).
